# Development of a Genus-Universal Nucleotide Signature for the Identification and Supervision of *Ephedra*-Containing Products

**DOI:** 10.3390/molecules27072342

**Published:** 2022-04-06

**Authors:** Gang Wang, Xuanjiao Bai, Xiaochen Chen, Ying Ren, Jianping Han

**Affiliations:** Institute of Medicinal Plant Development, Chinese Academy of Medical Sciences & Peking Union Medical College, Beijing 100193, China; wanggang@implad.ac.cn (G.W.); baixjz@163.com (X.B.); chloe.chen0802@gmail.com (X.C.); renying@implad.ac.cn (Y.R.)

**Keywords:** *Ephedra*, ephedrine alkaloids, methamphetamine, supervision, nucleotide signature, DNA barcoding

## Abstract

*Ephedra* plants generally contain ephedrine alkaloids, which are the critical precursor compounds of methamphetamine (METH). METH could cause serious physical and mental damage, and therefore *Ephedra* materials are strictly in supervision internationally. However, unlawful utilization of *Ephedra* herbs and its products still exist. Thus, it is imperative to establish a universal method for monitoring *Ephedra* ingredients in complex mixtures and processed products. In this study, 224 ITS2 sequences representing 59 taxa within *Ephedra* were collected, and a 23-bp genus-level nucleotide signature (GTCCGGTCCGCCTCGGCGGTGCG) was developed for the identification of the whole genus. The specific primers MH-1F/1R were designed, and 125 individuals of twelve *Ephedra* species/varieties were gathered for applicability verification of the nucleotide signature. Additionally, seven batches of Chinese patent medicines containing *Ephedra* herbs were used to test the application of the nucleotide signature in complex and highly processed materials. The results demonstrated that the 23-bp molecular marker was unique to *Ephedra* and conserved within the genus. It can be successfully utilized for the detection of *Ephedra* components in complex preparations and processed products with severe DNA degradation. The method developed in this study could undoubtedly serve as a strong support for the supervision of illegal circulation of *Ephedra*-containing products.

## 1. Introduction

*Ephedra* L. (Ephedraceae) contains approximately 67 species, mainly distributed in arid and desert regions of Asia, America, Europe, and Northern Africa [1]. In China, there are about 15 species and 4 varieties, some of which have been used as Chinese traditional medicine for a long history [2]. *Ephedra* herbs show satisfactory pharmacological activities in the treatment of cold, fever, asthma, flu, chills, headache, nasal congestion, and cough. Generally they are called “Ma Huang” and commonly found existing in Chinese classical prescriptions [3]. Modern pharmacological studies have also indicated that the crude extracts and isolated compounds from *Ephedra* have the effects of anti-inflammatory, antibacterial, antioxidant, anticancer, and hepatoprotection [4,5]. Classified as herbal supplements in the Dietary Supplement Health and Education Act of 1994, ephedra-containing products were largely unregulated without standardization in strength or purity. Between the 1990s and early 2000s, *Ephedra* ingredients were prevalent in dietary supplements and drugs for weight loss and physical performance enhancement [6]. It can accelerate metabolism and fatigue remission in bodies and can alter the intestinal flora of obese people [7]. However, afterwards, high-dose and long-term use of *Ephedra* materials was reported to cause a series of adverse effects due to the content of ephedrine-type alkaloids [8]. Ephedrine alkaloids, such as *l*-ephedrine, *d*-pseudoephedrine, *l*-methylephedrine, and *l*-norephedrine, are considered as the main active chemical constituents of *Ephedra* herb, but the misuse and abuse may induce the occurrence of hypertension, insomnia, anxiety, palpitations, arrhythmia, and even sudden death [9,10]. The data from the National Poison Data System (NPDS) of the United States showed that in 2002 alone, the number of the cases of ephedra poisoning reached up to 10,326, including 108 major effects and 3 deaths [11]. Thus, in 2004, the U.S. Food and Drug Administration (FDA) announced the prohibition of the sale of *Ephedra* and ephedrine-containing products, and the ban is still upheld [12]. However, the investigation of 105 weight loss products sold online in 2018 revealed that nearly 20% of the samples contained ephedrine alkaloids or *Ephedra* herb, which violated the 2004 ban of FDA and posed health and safety risks to individuals [13].

More worryingly, *Ephedra* species generally contain ephedrine alkaloids, which are the pivotal chemical precursors for the synthesis of methamphetamine, commonly known as “ice drug”. *Ephedra* herbs and related compound preparations have become the main raw materials for methamphetamine production by criminals [14]. The report on the Drug Situation in China [15], released by the Office of National Narcotics Control Commission, pointed out that methamphetamine was the most commonly abused drug in China, causing extremely serious damage to human’s physical and mental health. The government has imposed strict controls on the production, processing, circulation, and sale of *Ephedra* herb and its products. Nevertheless, illicit trading activities in the market still exist, with illegal elements extracting ephedrine from herb or compound preparations for the production of methamphetamine [16]. It is known that about 0.9 g of pseudoephedrine can be extracted from a box of New Contac capsules, and a bottle (100 mL) of Compound Furacilin Nasal Drops contains 0.1 g of ephedrine, while 0.75 g methamphetamine can be produced from 1 g ephedrine [17]. At present, pharmacies within China have set a purchase quantity limit of ephedrine-containing compound preparations and also require real-name registration of customers. However, to avoid inspection authorities, drug makers often prepare *Ephedra* materials in the form of powder, mixture, and crude extract, which leads to difficulties in their identification and brings challenges to customs inspection and market circulation supervision [18]. Therefore, it is necessary to develop a method for the accurate detection of mixture samples, compound preparations, and highly processed products containing *Ephedra* herb.

HPLC (high performance liquid chromatography) has been used in ephedrine alkaloids analysis, and SDS (sodium dodecyl sulfate) is often applied into the mobile phase to improve the resolution. However, it also brings more difficulties to separate the amphiphilic compounds. Lacking a specific and strong chromophore for chemical detection is also a challenge for conventional HPLC-UV detectors. The GC (gas chromatography) method is considered as the most popular technique for the quantitation of ephedrine analogs. For samples with complex composition, to enhance the sensitivity and remove interference compounds, tedious cleanup procedures and precolumn derivatization are required, resulting in pretty time-consuming protocols. Therefore, other more feasible and rapid methods may be needed for the detection of ephedrine or *Ephedra* herb-containing products. DNA barcoding has been recognized as a relatively simple and universal tool for species identification [19]. Chen et al. proposed the preliminary system of DNA barcoding for herbal materials based on the combination of internal transcribed spacer 2 (ITS2) and *psbA-trnH* barcodes, and it has been widely used in the authentication of medical plants [20]. However, the universal barcode sequences are often unsuitable for complex materials or deeply processed products due to severe DNA degradation. Shaw et al. found that short DNA fragments (88–121 bp) could be successfully amplified from pulverized samples that had been boiled for 60 min, while the longer sequences failed [21]. Meusnier et al. presented a “mini-barcode” to well address this problem. It was indicated that the full-length *CO1* with 650 bp could identify 97% of the tested species, while the identification success rates using the shorter sequences of 100 bp and 150 bp within *CO1* region also achieved 90% and 95%, respectively [22]. Furthermore, Han et al. put forward the nucleotide signature, generally 20–50 bp, for the determination of specific species in extracts, decoctions, and Chinese patent medicines [23,24,25]. *Ephedra* genus is the representative group containing ephedrine alkaloids and is the mono-genus belonging to Ephedraceae. Thus, it might be also feasible to develop a genus-level molecular marker for *Ephedra* L.

In the study, we aimed to develop a short molecular marker for the identification of *Ephedra* herb and its processed products. A unique 23-bp nucleotide signature (GTCCGGTCCGCCTCGGCGGTGCG) was found highly-conserved and universal within the whole *Ephedra* genus. Further, the genus-universal nucleotide signature was successfully applied to the determination of *Ephedra* component in compound preparations with severe DNA degradation. The method developed in our study can also serve as an adjunct to chemical analysis. It can help trace the source of ephedrine in ephedrine-containing products especially in forensic science and determine whether it is caused by the presence of *Ephedra* herbs. This study will undoubtedly provide a strong support for the supervision of *Ephedra*-containing products.

## 2. Materials and Methods

### 2.1. Collection of Materials

A total of 224 sequences of internal transcribed spacer 2 (ITS2) representing 59 taxa in *Ephedra* were downloaded from GenBank (https://www.ncbi.nlm.nih.gov/genbank/) (accessed on 19 August 2021) to develop the nucleotide signature. The accession numbers of downloaded sequences were shown in Table S1. To further verify the nucleotide signature, 125 individuals of 12 *Ephedra* species and varieties were collected from all over China (Table 1). The voucher specimens were deposited in the Herbarium of the Institute of Medicinal Plant Development, Chinese Academy of Medical Sciences, Beijing, China. To evaluate the detection ability of this method, seven batches of Chinese patent medicines containing *Ephedra* herb (called Ephedrae Herba in formulas) were purchased from drug stores in Beijing, Heilongjiang, Shandong, and Guangdong provinces, in the forms of tablets, honeyed pills, watered pills, and concentrated pills (Table 2).

### 2.2. DNA Extraction

Voucher samples: The surface of specimen samples was cleaned up with 75% ethanol, and then approximately 20 mg of each sample was added to a centrifuge tube for DNA extraction. The samples were ground into fine powder with a ball-milling instrument (Retsch Co., Shanghai, China) at a frequency of 30 Hz for 2 min. Then the genomic DNA was extracted using a Plant Genomic DNA Extraction Kit (Tiangen Biotech Beijing Co., Beijing, China) according to manufacturer’s instructions.

Chinese patent medicines: Approximately 50 mg of the Chinese patent medicines was added to a centrifuge tube, and six parallel tubes of each preparation were prepared. Seven-hundred microliters of the prewash buffer (700 mM NaCl; 100 mM Tris-HCl, pH 8.0; 20 mM EDTA, pH 8.0; 2% PVP-40; and 0.4% β-mercaptoethanol) was added to the tubes to wash the powder several times until the supernatant was colorless and transparent, and then the mixture was centrifuged at 7500× *g* for 5 min. The genomic DNA was subsequently extracted from the precipitate via Plant Genomic DNA Extraction Kit (Tiangen Biotech Beijing Co.) in accordance with manufacturer’s instructions. Finally, the DNA in six replicates of the same batch was eluted with 50 μL of double-distilled water into a single tube. The DNA quality of voucher samples and Chinese patent medicines was examined via Nanodrop 2000 (Thermo Scientific, Waltham, Massachusetts, USA). The value of A_260/280_ was 1.8–2.0, and the DNA concentration ranged from 60 ng/μL to 80 ng/μL, which met the requirements of subsequent PCR reaction.

### 2.3. Primer Design, PCR Amplification and Sequencing

The primer pair MH-1F (5′-TCATCGAGTCTTTGAACGC-3′)/MH-1R (5′- ATGCGAAGGTCCCCTTTT-3′) was designed using Primer Premier 6.0 software (Premier Co., Palo Alto, CA, USA) for the amplification of the short DNA fragments (~150 bp) containing the nucleotide signature of *Ephedra* L. Polymerase chain reactions (PCR) were performed in a 25-µL system consisting of 12.5 µL of 2 × PCR Master Mix (Aidlab Biotechnologies Co., Beijing, China), 1.0 μL of forward/reverse primers (MH-1F/MH-1R, 2.5 μM), and 2.0 μL of DNA templates and filled with double-distilled water. The reactions were then performed as follows: 94 °C for 5 min; followed by 35 cycles of 94 °C for 45 s, 56 °C for 1 min, and 72 °C for 1 min; and a final extension at 72 °C for 10 min. The PCR products were examined via 1% (*w*/*v*) agarose gel electrophoresis that had been prestained by GelRed (Mei5 Biotechnology Co., Beijing, China) and bidirectionally sequenced using an ABI 3730XL sequencer (Applied Biosystems Co., Foster City, CA, USA) at the Major Engineering laboratory of Chinese Academy of Agricultural Sciences (Beijing, China).

### 2.4. Sequence Analysis

The relatively conserved regions, screened out from downloaded ITS2 sequences of *Ephedra* L, were selected as the candidates of the nucleotide signature. BLAST analysis was carried out in the website of National Center for Biotechnology Information (NCBI, https://www.ncbi.nlm.nih.gov/) (accessed on 19 August 202) for the validation of the intragenus conservation and intergeneric specificity of the nucleotide signature. The sequencing data of voucher specimens and Chinese patent medicine were edited and assembled via CodonCode Aligner 5.2.0 (CodonCode Co., Centerville, MA, USA). The sequences were then aligned by MEGA-X software. All the experimental sequences of the 125 specimens were submitted to GenBank database, with accession numbers listed in Table 1.

## 3. Results

### 3.1. Development of the Genus-Universal Nucleotide Signature for Ephedra L.

Through sequence screening of 224 ITS2 sequences representing 59 taxa downloaded from GenBank, a 55-bp relatively conserved fragment was obtained as the candidate of the nucleotide signature of *Epedra* L. The regions with different lengths within the selected fragment were intercepted and BLAST analysis was performed to determine the optimal sequence size of the nucleotide signature. It was indicated that longer DNA segments were less conserved in genus level, and a single sequence cannot cover the entire *Ephedra* L. (Figures S1–S5). However, too short sequences lost their intergeneric specificity and matched the species outside *Ephedra* genus (Figure S6). Finally, a 23-bp fragment (GTCCGGTCCGCCTCGGCGGTGCG) was identified as the unique nucleotide signature for *Ephedra* (Table 3). This sequence was highly conservative in *Ephedra* L., and no mutation sites were found in the nucleotide signature region of the 224 downloaded ITS2 sequences. BLAST results in NCBI also showed that the 23-bp nucleotide signature was specific to *Ephedra* genus, and there was at least one variation in this region between *Ephedra* and non-*Ephedra* (Figure S7). Therefore, the 23-bp nucleotide signature can be considered as a genus-universal molecular marker for *Ephedra* L.

### 3.2. Verification of the Nucleotide Signature in the Species and Varieties within Ephedra L.

**Figure 1:**
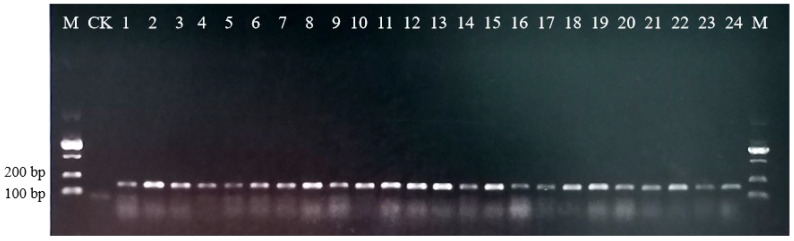
Amplification of 12 species and varieties in *Ephedra* genus with the primers MH-1F/MH-1R. M. DNA marker; CK. Negative control; 1–3. *E. equisetina*; 4. *E. fedtschenkoae*; 5–6. *E. gerardiana*; 7–9. *E. intermedia*; 10–13. *E. intermedia var. tibetica*; 14. *E. likiangensis*; 15. *E. minuta*; 16. *E. monosperma*; 17–18. *E. przewalskii*; 19. *E. regeliana*; 20–21. *E. saxatilis*; 22–24. *E. sinica*.

To further test the reliability and universality of the nucleotide signature in *Ephedra* L., 125 individuals covering 12 species and varieties were collected. The primer pair MH-1F/MH-1R was designed to amplify the short DNA fragments containing the nucleotide signature. The results showed that all specimens were successfully amplified, indicating a good applicability of the primers (Figure 1). Finally, 125 sequences of 152–153 bp were obtained from specimen samples, which were subsequently aligned with the 224 ITS2 sequences of *Ephedra* L. downloaded from GenBank. The alignment of the total 349 ITS2 regions demonstrated that the 23-bp nucleotide signature existed in all the sequences without any variations. In conclusion, the developed nucleotide signature was universal within *Ephedra* L. and could be amplified successfully from specimens by using the primers MH-1F/MH-1R.

### 3.3. Application of the Nucleotide Signature on the Detection of Ephedra Herb in Compound Preparations

**Figure 2:**
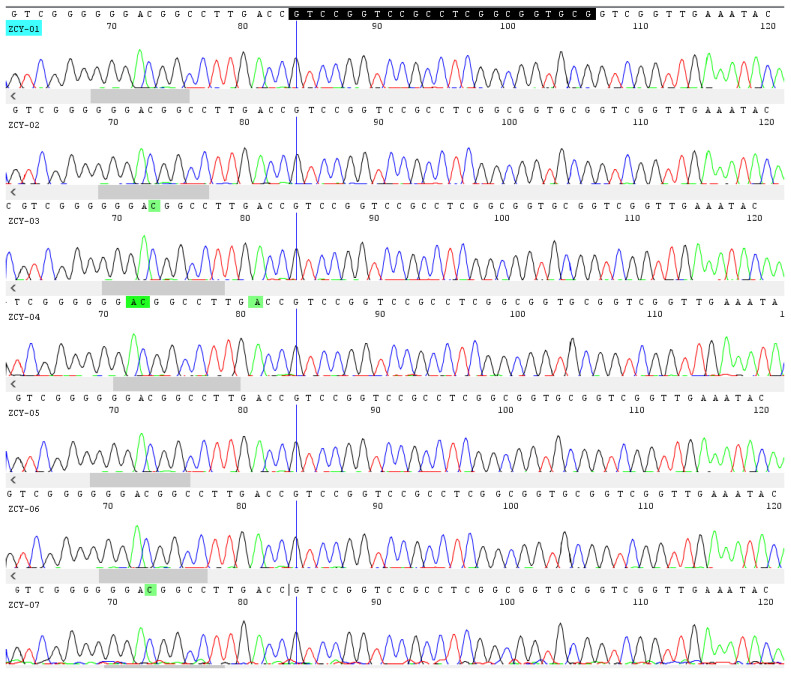
Sequencing peak profiles of seven batches of Chinese patent medicine containing *Ephedra* herb.

To validate the application of this method on complex and processed products, a total of seven batches of commercial Chinese patent medicines containing *Ephedra* herb were gathered from pharmacies in China, i.e., Mahuang Fuzi Xixin Soup, Maxing Zhike Tablets, Mahuang Zhisou Pills, Tongxuan Lifei Pills, Xiaoer Feire Kechuan Granules, Zhengtian Pills, and Qiguanyan Pills. The formulations of these preparations consisted of 3 (Mahuang Fuzi Xixin Soup) to 31 (Qiguanyan Pills) ingredients, respectively (Table 2). All the compound preparations were successfully amplified and sequenced using the designed primers MH-1F/MH-1R. It was showed that the *Ephedra* herb listed on label could be detected in the seven Chinese patent medicine samples by retrieving the nucleotide signature in sequencing results. Moreover, the direct sequencing of the PCR products presented very clean traces (Figure 2), which illustrated that the MH-1F/1R primer pair could specifically amplify the nucleotide signature of *Ephedra* in a complex system. To sum up, the nucleotide signature together with specific primers was suitable for the inspection of *Ephedra* ingredients in compound preparations and other deeply processed products.

## 4. Discussion

### 4.1. Significance of the Development of a Nucleotide Signature for Ephedra

DNA barcoding is a powerful tool for species authentication based on species-specific differences of a standard and short DNA sequence. It can help achieve the rapid, accurate, and automated identification of plants and animals [26,27]. At present, a universal standard as well as the databases for DNA barcoding have been established and widely applied [28]. It was also suggested as an efficient tool for the supervision of herbal markets. Han et al. investigated 1436 samples representing 295 medicinal species from seven herb markets in China, and found that 1260 samples were successfully identified by ITS2 barcodes, among which approximately 4.2% were adulterants [29]. The survey of species authenticity for herbal products using DNA barcoding showed that only 62% of the 21 samples labeled as “Nan-she-teng (*Celastrus orbiculatus*)” and 31% of the 26 samples labeled as “Lei-gong-teng (*Tripterygium wilfordii*)” were genuine [30]. However, in view of the strict regulation, to avoid inspection authorities, the *Ephedra* raw materials are often transported and traded by lawbreakers in the forms of powder, crude extract, compound preparations, and other processed products. In this circumstance, conventional DNA barcodes are no longer applicable due to severe DNA degradation. Liu et al. found that complete ITS2 region could not be amplified from herbal decoction using universal primers 2F/3R [23]. Song et al. also indicated that the five primary barcode loci (ITS2, *psbA-trnH*, *rbcL*, *matK*, and *trnL*) could be successfully amplified in only 8.89%–20% of processed samples, while the amplification rate of the short *trnL* (UAA) intron P6 loop was up to 75.56% [31]. Thus, in this study, we aimed to develop a short nucleotide signature to more effectively identify *Ephedra*-containing products.

An ideal nucleotide signature at genus level should meet the qualifications of both intragenus conservation and intergenus specificity. In this study, 224 ITS2 sequences of 59 taxa in *Ephedra* L. were downloaded from GenBank to develop a 23-bp nucleotide signature. Then 125 individuals representing 12 *Ephedra* species and varieties were collected together to test the nucleotide signature. In total, nearly 90% of the taxa within *Ephedra* genus were involved. Considering the possible errors in published data, firstly we verified the accuracy of the sequences downloaded from GenBank by BLAST analysis to ensure their reliability. Finally, it was proved that the 23-bp nucleotide signature universally indeed existed in *Ephedra* L. The 125 experiment specimens, collected from different areas in China, were identified by professional taxonomists, and the nucleotide signature was found to exist in all the samples without any mutations. It can be inferred that the nucleotide signature is ubiquitous and highly conserved in *Ephedra* genus. In addition, the BLAST results in NCBI also showed that the nucleotide signature was unique to *Ephedra* L. Therefore, we believe that currently available data is sufficient to support that the 23-bp DNA fragment can serve as a molecular tag for the identification of *Ephedra*-containing products. Additionally, compared to species-specific molecular markers, the genus-level nucleotide signature can effectively avoid the complex work of developing multiple detection methods for different taxa in *Ephedra*.

### 4.2. Supervision of Ephedra-Containing Products with the Genus-Universal Nucleotide Signature

The genus *Ephedra* contains about 67 species widely distributed around the world. The chemical constituents in *Ephedra* generally include alkaloids, flavonoids, tannins, polysaccharides, phenolics, etc [32], among which ephedrine-type alkaloids are regarded as the main active ingredients to exert pharmacological effects [33]. *Ephedra* herb has been used in traditional Chinese medicine with a long history, which was originally recorded in the ancient book of *Shennong’s Classic of Materia Medica*. More than 60 classical prescriptions containing *Ephedra* herb are recorded in the Chinese Pharmacopoeia (2020 edition). Besides officinal value, *Ephedra* materials were also supplemented into dietary, health care products, and cosmetics for weight loss and energy enhancement [34]. However, under the unrestricted and controlled use, more and more side effects on people appeared continuously [35]. The subsequent studies found that long-term or high-dose use of ephedrine-containing products can lead to hypertension, arrhythmia, stroke, cardiac arrest, and even sudden death [36]. Though the government and regulatory authorities have strictly controlled *Ephedra* and ephedrine products, its illegal production and sales still exist. Worryingly, ephedrine is also an important raw material for the production of methamphetamine. Ephedrine obtained by criminals from *Ephedra* herbs, crude extracts, and compound preparations for the synthesis of methamphetamine have caused great damage to society and human’s physical and mental health [18]. In a survey of 592 drug users, more than 70% claimed to have injected methamphetamine, and the proportion of the participants reporting methamphetamine as their most frequently injected drug increased from 2.1% in 2005 to 29.6% in 2015 [37]. Although chemical methods such as HPLC and GC have been widely used for the detection of ephedrine and *Ephedra* herbs, for processed samples with complex components, the interfering components will affect the analysis results, and the tedious pre-treatment process will greatly increase the detection time. Thus, it is urgent to establish other more effective methods for the supervision of *Ephedra* and its deeply processed products.

In this study, a 23-bp nucleotide signature was developed for the detection of *Ephedra* herb and its products with severely degraded DNA, which has been proved to be conserved within *Ephedra* genus and divergent with other genera. Furthermore, the method was applied to the determination of seven batches of Chinese patent medicines that contained *Ephedra* ingredients. The results showed that *Ephedra* herb could be successfully detected in all the samples by seeking the nucleotide signature in sequencing results. It is worth emphasizing that in the proprietary Chinese patent medicine Qiguanyan Pills, in addition to Ephedrae Herba, there are an additional 30 kinds of other herbs, including Armeniacae Semen Amarum (Kuxingren), Gypsum Fibrosum (Shigao), Glycyrrhizae Radix Et Rhizoma (Gancao), Peucedani Radix (Qianhu), Cynanchi Stauntonii Rhizoma Et Radix (Baiqian), Stemonae Radix (Baibu), etc. The nucleotide signature could be still amplified and sequenced successfully with the newly designed primers MH-1F/MH-1R. It is suggested that even if the ingredients are complex and diverse, the *Ephedra* herb can still be detected, which simultaneously reflects the high sensitivity of this method. In conclusion, the genus-universal nucleotide signature proposed in this study can be considered as a powerful tool for the supervision of *Ephedra* and ephedrine-containing products.

## 5. Conclusions

In this study, a 23-bp genus-specific nucleotide signature was developed for the detection of *Ephedra* herb and its highly processed products. The nucleotide signature was verified to be unique and highly conserved within *Ephedra*, which could be successfully amplified and sequenced from processed products containing *Ephedra* ingredients using the primer pair MH-1F/MH-1R. This method can also serve as an adjunct to chemical analysis to help trace the source of ephedrine in ephedrine-containing products especially in forensic science. Besides, the genus-level molecular marker effectively avoids the tedious work of establishing various identification/detection methods for different species in *Ephedra*. Our study will undoubtedly provide a strong support for the identification and regulation of *Ephedra*-containing products to prevent illegal supplement and methamphetamine production.

## Figures and Tables

**Table 1:** Information of the *Ephedra* samples in this study.

No.	Species	Voucher No.	Collection Site	GenBank Accession
1	*E. equisetina*	IMD0001954	Hebei	OL456774
2	*E. equisetina*	IMD0001953	Shaanxi	OL456770
3	*E. equisetina*	IMD0001955	Shanxi	OL456771
4	*E. equisetina*	IMD0001952	Hebei	OL456772
5	*E. equisetina*	IMD0001951	Xinjiang	OL456773
6	*E. equisetina*	IMD0001950	Xinjiang	OL456775
7	*E. equisetina*	IMD0001949	Xinjiang	OL456776
8	*E. equisetina*	IMD0001946	Xinjiang	OL456777
9	*E. equisetina*	IMD0001964	Beijing	OL456763
10	*E. equisetina*	IMD0001963	Beijing	OL456764
11	*E. equisetina*	IMD0001966	Xinjiang	OL456765
12	*E. equisetina*	IMD0001961	Hebei	OL456766
13	*E. equisetina*	IMD0001958	Jiangsu	OL456767
14	*E. equisetina*	IMD0001957	Beijing	OL456768
15	*E. equisetina*	IMD0001956	Beijing	OL456769
16	*E. fedtschenkoae*	IMD0001967	Qinghai	OL456778
17	*E. gerardiana*	IMD0001970	Xizang	OL456779
18	*E. gerardiana*	IMD0001969	Xizang	OL456780
19	*E. gerardiana*	IMD0001968	Xizang	OL456781
20	*E. gerardiana*	IMD0001971	Xizang	OL456782
21	*E. gerardiana*	IMD0001972	Xizang	OL456783
22	*E. intermedia*	IMD0001980	Qinghai	OL456784
23	*E. intermedia*	IMD0001978	Qinghai	OL456785
24	*E. intermedia*	IMD0001976	Xinjiang	OL456786
25	*E. intermedia*	IMD0001975	Qinghai	OL456787
26	*E. intermedia*	IMD0001974	Xinjiang	OL456788
27	*E. intermedia*	IMD0001995	Sichuan	OL456789
28	*E. intermedia*	IMD0001994	Neimenggu	OL456790
29	*E. intermedia*	IMD0001992	Xinjiang	OL456791
30	*E. intermedia*	IMD0001991	Xinjiang	OL456792
31	*E. intermedia*	IMD0001989	Xinjiang	OL456793
32	*E. intermedia*	IMD0001988	Hebei	OL456794
33	*E. intermedia*	IMD0001996	Neimenggu	OL456795
34	*E. intermedia*	IMD0001986	Xinjiang	OL456796
35	*E. intermedia*	IMD0001984	Gansu	OL456797
36	*E. intermedia*	IMD0001983	Xinjiang	OL456798
37	*E. intermedia*	IMD0001981	Gansu	OL456799
38	*E. intermedia*	IMD0001985	Sichuan	OL456800
39	*E. intermedia*	IMD0001987	Xinjiang	OL456801
40	*E. intermedia* var. *tibetica*	IMD0001982	Xizang	OL456802
41	*E. intermedia* var. *tibetica*	IMD0002003	Xizang	OL456803
42	*E. intermedia* var. *tibetica*	IMD0002002	Xizang	OL456804
43	*E. intermedia* var. *tibetica*	IMD0002001	Xizang	OL456805
44	*E. intermedia* var. *tibetica*	IMD0002000	Xizang	OL456806
45	*E. intermedia* var. *tibetica*	IMD0001999	Xizang	OL456807
46	*E. intermedia* var. *tibetica*	IMD0001998	Xizang	OL456808
47	*E. intermedia* var. *tibetica*	IMD0001997	Xizang	OL456809
48	*E. intermedia* var. *tibetica*	IMD0002016	Xizang	OL456810
49	*E. intermedia* var. *tibetica*	IMD0002014	Xizang	OL456811
50	*E. intermedia* var. *tibetica*	IMD0002013	Xizang	OL456812
51	*E. intermedia* var. *tibetica*	IMD0002012	Xizang	OL456813
52	*E. intermedia* var. *tibetica*	IMD0002011	Xizang	OL456814
53	*E. intermedia* var. *tibetica*	IMD0002010	Xizang	OL456815
54	*E. intermedia* var. *tibetica*	IMD0002009	Xizang	OL456816
55	*E. intermedia* var. *tibetica*	IMD0002007	Xizang	OL456817
56	*E. intermedia* var. *tibetica*	IMD0002006	Xizang	OL456818
57	*E. intermedia* var. *tibetica*	IMD0002005	Xizang	OL456819
58	*E. intermedia* var. *tibetica*	IMD0002008	Xizang	OL456820
59	*E. intermedia* var. *tibetica*	IMD0002021	Xizang	OL456821
60	*E. intermedia* var. *tibetica*	IMD0002017	Xizang	OL456822
61	*E. intermedia* var. *tibetica*	IMD0002019	Xizang	OL456823
62	*E. intermedia* var. *tibetica*	IMD0002020	Xizang	OL456824
63	*E. intermedia* var. *tibetica*	IMD0002018	Xizang	OL456825
64	*E. intermedia* var. *tibetica*	IMD0002024	Xizang	OL456826
65	*E. intermedia* var. *tibetica*	IMD0002023	Xizang	OL456827
66	*E. intermedia* var. *tibetica*	IMD0002022	Xizang	OL456828
67	*E. likiangensis*	IMD0002027	Yunnan	OL456829
68	*E. likiangensis*	IMD0002026	Sichuan	OL456830
69	*E. likiangensis*	IMD0002025	Yunnan	OL456831
70	*E. minuta*	IMD0002029	Xizang	OL456832
71	*E. minuta*	IMD0002030	Xizang	OL456833
72	*E. minuta*	IMD0002028	Sichuan	OL456834
73	*E. monosperma*	IMD0002037	Qinghai	OL456835
74	*E. monosperma*	IMD0002043	Xizang	OL456836
75	*E. monosperma*	IMD0002044	Xizang	OL456837
76	*E. przewalskii*	IMD0002060	Gansu	OL456841
77	*E. przewalskii*	IMD0002059	Qinghai	OL456842
78	*E. przewalskii*	IMD0002057	Gansu	OL456843
79	*E. przewalskii*	IMD0002056	Xinjiang	OL456844
80	*E. przewalskii*	IMD0002055	Qinghai	OL456845
81	*E. przewalskii*	IMD0002063	Gansu	OL456846
82	*E. przewalskii*	IMD0002062	Gansu	OL456838
83	*E. przewalskii*	IMD0002061	Gansu	OL456839
84	*E. przewalskii*	IMD0002051	Xinjiang	OL456840
85	*E. regeliana*	IMD0002066	Qinghai	OL456850
86	*E. regeliana*	IMD0002064	Xinjiang	OL456847
87	*E. regeliana*	IMD0002067	Qinghai	OL456848
88	*E. regeliana*	IMD0002070	Xinjiang	OL456849
89	*E. saxatilis*	IMD0002071	Xizang	OL456851
90	*E. saxatilis*	IMD0002078	Xizang	OL456852
91	*E. saxatilis*	IMD0002076	Xizang	OL456853
92	*E. saxatilis*	IMD0002075	Xizang	OL456854
93	*E. saxatilis*	IMD0002077	Sichuan	OL456855
94	*E. saxatilis*	IMD0002081	Xizang	OL456856
95	*E. saxatilis*	IMD0002080	Xizang	OL456857
96	*E. saxatilis*	IMD0002079	Xizang	OL456858
97	*E. saxatilis*	IMD0002090	Xizang	OL456859
98	*E. saxatilis*	IMD0002089	Xizang	OL456860
99	*E. saxatilis*	IMD0002088	Xizang	OL456861
100	*E. saxatilis*	IMD0002087	Xizang	OL456862
101	*E. sinica*	IMD0002098	Hebei	OL456887
102	*E. sinica*	IMD0002099	Hebei	OL456863
103	*E. sinica*	IMD0002097	Hebei	OL456864
104	*E. sinica*	IMD0002095	Shanxi	OL456865
105	*E. sinica*	IMD0002106	Hebei	OL456866
106	*E. sinica*	IMD0002105	Neimenggu	OL456867
107	*E. sinica*	IMD0002104	Hebei	OL456868
108	*E. sinica*	IMD0002103	Hebei	OL456869
109	*E. sinica*	IMD0002101	Shanxi	OL456870
110	*E. sinica*	IMD0002109	Neimenggu	OL456871
111	*E. sinica*	IMD0002108	Hebei	OL456872
112	*E. sinica*	IMD0002118	Neimenggu	OL456873
113	*E. sinica*	IMD0002117	Neimenggu	OL456874
114	*E. sinica*	IMD0002115	Neimenggu	OL456875
115	*E. sinica*	IMD0002112	Neimenggu	OL456876
116	*E. sinica*	IMD0002111	Jilin	OL456877
117	*E. sinica*	IMD0002125	Neimenggu	OL456878
118	*E. sinica*	IMD0002122	Neimenggu	OL456879
119	*E. sinica*	IMD0002123	Neimenggu	OL456880
120	*E. sinica*	IMD0002121	Neimenggu	OL456881
121	*E. sinica*	IMD0002119	Hebei	OL456882
122	*E. sinica*	IMD0002136	Neimenggu	OL456883
123	*E. sinica*	IMD0002135	Neimenggu	OL456884
124	*E. sinica*	IMD0002134	Neimenggu	OL456885
125	*E. sinica*	IMD0002132	Neimenggu	OL456886

**Table 2:** Detailed information of the seven batches of Chinese patent medicine.

No.	Sample Name	Type	Collection Site	Number of Ingredients	Ingredients on Label
ZCY-01	Mahuang Fuzi Xixin Soup	Concentrated pills	Guangdong	3	**Ephedrae Herba**, Aconiti Lateralis Radix Praeparata, Asari Radix Et Rhizoma
ZCY-02	Maxing Zhike Tablets	Tablets	Jilin	4	**Ephedrae Herba**, Armeniacae Semen Amarum, Gypsum Fibrosum, Glycyrrhizae Radix Et Rhizoma Praeparata Cum Melle
ZCY-03	Mahuang Zhisou Pills	Watered pills	Shandong	7	Citri Exocarpium Rubrum, **Ephedrae Herba**, Platycodonis Radix, Fritillariae Cirrhosae Bulbus, Schisandrae Chinensis Fructus, Poria, Asari Radix Et Rhizoma
ZCY-04	Tongxuan Lifei Pills	Honeyed pills	Beijing	11	Perillae Folium, Peucedani Radix, Platycodonis Radix, Armeniacae Semen Amarum, **Ephedrae Herba**, Glycyrrhizae Radix Et Rhizoma, Citri Reticulatae Pericarpium, Pinelliae Rhizoma, Poria, Aurantii Fructus, Scutellariae Radix
ZCY-05	Xiaoer Feire Kechuan Granules	Granules	Heilongjiang	11	**Ephedrae Herba**, Armeniacae Semen Amarum, Gypsum Fibrosum, Glycyrrhizae Radix Et Rhizoma, Lonicerae Japonicae Flos, Forsythiae Fructus, Anemarrhenae Rhizoma, Scutellariae Radix, Isatidis Radix, Ophiopogonis Radix, Houttuyniae Herba
ZCY-06	Zhengtian Pills	Watered pills	Guangdong	15	Uncariae Ramulus Cum Uncis, Paeoniae Radix Alba, Chuanxiong Rhizoma, Angelicae Sinensis Radix, Rehmanniae Radix, Angelicae Dahuricae Radix, Saposhnikoviae Radix, Notopterygii Rhizoma Et Radix, Persicae Semen, Carthami Flos, Asari Radix Et Rhizoma, Angelicae Pubescentis Radix, **Ephedrae Herba**, Aconiti Lateralis Radix Praeparata, Spatholobi Caulis
ZCY-07	Qiguanyan Pills	Watered pills	Beijing	31	**Ephedrae Herba**, Armeniacae Semen Amarum, Gypsum Fibrosum, Glycyrrhizae Radix Et Rhizoma, Peucedani Radix, Cynanchi Stauntonii Rhizoma Et Radix, Stemonae Radix, Asteris Radix Et Rhizoma, Farfarae Flos, Meretricis Concha Cyclinae Concha, Descurainiae Semen Lepidii Semen, Citri Grandis Exocarpium, Platycodonis Radix, Poria, Pinelliae Rhizoma, Polygalae Radix, Inulae Flos, Pumice, Perillae Fructus, Codonopsis Radix, Jujubae Fructus, Schisandrae Chinensis Fructus, Cinnamomi Ramulus, Allii Macrostemonis Bulbus, Paeoniae Radix Alba, Mori Folium, Belamcandae Rhizoma, Scutellariae Radix, Indigo Naturalis, Taraxaci Herba, Eriobotryae Folium

**Table 3:** BLAST results of the candidate sequences with different lengths in NCBI.

No.	Sequences (5′-3′)	Length/bp	Intergeneric Specificity	Intragenus Conservation
S1	TCGGGGGGACGGCCTTGACCGTCCGGTCCGCCTCGGCGGTGCGGTCGGTTGAAAT	55	√	×
S2	GGGGGACGGCCTTGACCGTCCGGTCCGCCTCGGCGGTGCGGTCGG	45	√	×
S3	GCCTTGACCGTCCGGTCCGCCTCGGCGGTGCGGTC	35	√	×
S4	GACCGTCCGGTCCGCCTCGGCGGTGCGGTC	30	√	×
S5	CCGTCCGGTCCGCCTCGGCGGTGCGGT	27	√	×
**S6**	**GTCCGGTCCGCCTCGGCGGTGCG**	**23**	**√**	**√**
S7	CCGGTCCGCCTCGGCGGTGC	20	×	√

## Data Availability

The data presented in this study are openly available in https://www.ncbi.nlm.nih.gov/ with the GenBamk accession numbers in Table 1.

## Supplementary Materials

The following supporting information can be downloaded at: https://www.mdpi.com/article/10.3390/molecules27072342/s1, Table S1: Information of the 224 ITS2 sequences of *Ephedra* downloaded from GenBank; Figure S1: BLAST results in NCBI of the sequence S1; Figure S2: BLAST results in NCBI of the sequence S2; Figure S3: BLAST results in NCBI of the sequence S3; Figure S4: BLAST results in NCBI of the sequence S4; Figure S5: BLAST results in NCBI of the sequence S5; Figure S6: BLAST results in NCBI of the sequence S7; Figure S7: BLAST results in NCBI of the nucleotide signature of *Ephedra* L.
