# Supplementary material for: Development of a Genus-Universal Nucleotide Signature for the Identification and Supervision of Ephedra-Containing Products
Source: Molecules. 2022 Apr 6;27(7):2342. doi: 10.3390/molecules27072342 (PMC9000813; doi:10.3390/molecules27072342)
Supplement: Supplementary file 1 [file molecules-27-02342-s001.zip › molecules-1664713-supplementary.pdf]

Supplementary Materials:

Supplementary Tables:

Table S1. Information of the 224 ITS2 sequences of *Ephedra* downloaded from GenBank.

Supplementary Figures:

Figure S1. BLAST results in NCBI of the sequence S1.

Figure S2. BLAST results in NCBI of the sequence S2.

Figure S3. BLAST results in NCBI of the sequence S3.

Figure S4. BLAST results in NCBI of the sequence S4.

Figure S5. BLAST results in NCBI of the sequence S5.

Figure S6. BLAST results in NCBI of the sequence S7.

Figure S7. BLAST results in NCBI of the nucleotide signature of *Ephedra* L.

Table S1

No.	Species	GenBank accession
1	*Ephedra alata*	MH547540
2	*Ephedra altissima*	AY755772-73
3	*Ephedra americana*	GU968545, GU968567, KP788809
4	*Ephedra antisyphilitica*	AY755757, KP788810
5	*Ephedra aphylla*	AY755771, KP788811, MG569946, GU968544、52、69
6	*Ephedra aspera*	KP788812-13
7	*Ephedra boelckei*	KP788814
8	*Ephedra botschantzevii*	GU968565
9	*Ephedra breana*	KP788815
10	*Ephedra californica*	AY755750, KP788816
11	*Ephedra chilensis*	AY755754、67, KP788817
12	*Ephedra ciliata*	AY755776, GU968548
13	*Ephedra compacta*	KP788818-19
14	*Ephedra coryi*	KP788820-21
15	*Ephedra cutleri*	KP788822
16	*Ephedra dahurica*	GU186923、81-83, JQ726575
17	*Ephedra distachya*	AY755761、69, FJ958013, GU186924、68、71、73-74、76-78、83-84、93, KX779084, KP788823
18	*Ephedra distachya* subsp. *helvetica*	JQ726579
19	*Ephedra equisetina*	AY394065, AY755740、51、70, GQ434578-79, GU968572, KP788824, KX675037-39, KX779079-82, MF096243-48
20	*Ephedra fedtschenkoae*	KX779072
21	*Ephedra foeminea*	FJ958007, GU290485, GU968546、51, KP788808、25
22	*Ephedra foliata*	AY755775, FJ958008-09
23	*Ephedra fragilis*	AY755752, FJ958014
24	*Ephedra frustillata*	AY755743、48, KP788826
25	*Ephedra funerea*	KP788827-28
26	*Ephedra gerardiana*	AY394067, AY755745、53、60
27	*Ephedra gracilis*	KP788829
28	*Ephedra intermedia*	AY394062, AY755741、58, GU968563, KP788830, KX675040-42, KX779076、87-88, MF096249-51、53-55, MH258138、80-83
29	*Ephedra intermedia* var. *tibetica*	KX779085
30	*Ephedra likiangensis*	AY394068, AY755739、66, GU968547, KP788831, MF972970, JQ726581
31	*Ephedra lomatolepis*	FJ958006, GU290484, GU968562、64, JQ726585-86, KX779083
32	*Ephedra major*	AY755778, FJ958003
33	*Ephedra major* subsp. *major*	FJ958012, GU968557
34	*Ephedra major* subsp. *procera*	AY755763, FJ958011, GU968554-56、59, JQ726580、90, MG256238
35	*Ephedra minuta*	AY394069, AY755742、55-56、JQ726582
36	*Ephedra monosperma*	AY394066, AY755746, GU968560, KP788832, KX779073-75
37	*Ephedra multiflora*	KP788833
38	*Ephedra nevadensis*	AY755764

39	*Ephedra ochreata*	KP788834-35
40	*Ephedra pachyclada*	AY755779, MG569945
41	*Ephedra pedunculata*	GU968561, KP788836
42	*Ephedra przewalskii*	AY394064, AY730605-06, JQ726588, KX779077-78
43	*Ephedra pseudodistachya*	JQ726587、89, JQ726592
44	*Ephedra regeliana*	AY730607, KX779086
45	*Ephedra rhytidosperma*	DQ028782, DQ212958
46	*Ephedra rupestris*	AY755765, KP788837
47	*Ephedra sarcocarpa*	FJ958017
48	*Ephedra saxatilis*	FJ958022, MF785606, MN861080
49	*Ephedra sinica*	AY394063, AY755747、49, GQ865704, GU186922, GU968550, JF421494, KP788838, KX675043-45, KX779071, MF096252、56-60, MF972965-69
50	*Ephedra sinica* var. *pumila*	GU968566
51	*Ephedra somalensis*	FJ958004
52	*Ephedra strobilacea*	FJ958018
53	*Ephedra tilhoana*	JQ726591
54	*Ephedra torreyana*	AY755759, KP788839
55	*Ephedra transitoria*	FJ958021
56	*Ephedra triandra*	KP788840
57	*Ephedra trifurca*	AY755762, KP788841
58	*Ephedra tweediana*	AY755768, KP788842
59	*Ephedra viridis*	FJ958005, KP788843
60	*Ephedra* sp.	FJ958023, MH732928

Figure S1

Description	Max Score	Total Score	Query Cover	E value	Per. Ident	Acc. Len	Accession
Ephedra likiangensis voucher LJ01 internal transcribed spacer 2, partial sequence	102	102	100%	1e-18	100.00%	246	MF972970.1
Ephedra sinica voucher CM04 internal transcribed spacer 2, partial sequence	102	102	100%	1e-18	100.00%	261	MF972969.1
Ephedra sinica voucher CM03 internal transcribed spacer 2, partial sequence	102	102	100%	1e-18	100.00%	261	MF972968.1
Ephedra sinica voucher CM01 internal transcribed spacer 2, partial sequence	102	102	100%	1e-18	100.00%	261	MF972967.1
Ephedra sinica voucher CM05 internal transcribed spacer 2, partial sequence	102	102	100%	1e-18	100.00%	261	MF972966.1
Ephedra sinica voucher CM02 internal transcribed spacer 2, partial sequence	102	102	100%	1e-18	100.00%	261	MF972965.1
Ephedra major subsp. procera voucher 823-12-2014 5.8S ribosomal RNA gene, partial sequence; internal transcribed spacer 2, co...	102	102	100%	1e-18	100.00%	331	MG256238.1
Ephedra aphylla voucher SCUI-E13 5.8S ribosomal RNA gene, partial sequence; internal transcribed spacer 2, complete sequence...	102	102	100%	1e-18	100.00%	356	MG569946.1
Ephedra intermedia isolate XZZMH4 internal transcribed spacer 2, complete sequence	102	102	100%	1e-18	100.00%	251	MH258183.1
Ephedra intermedia isolate XZZMH3 internal transcribed spacer 2, complete sequence	102	102	100%	1e-18	100.00%	251	MH258182.1
Ephedra intermedia isolate XZZMH2 internal transcribed spacer 2, complete sequence	102	102	100%	1e-18	100.00%	251	MH258181.1
Ephedra intermedia isolate XZZMH1 internal transcribed spacer 2, complete sequence	102	102	100%	1e-18	100.00%	251	MH258180.1
Ephedra intermedia isolate XZZMH internal transcribed spacer 2, complete sequence	102	102	100%	1e-18	100.00%	251	MH258138.1
Ephedra alata isolate UQU ALP192 5.8S ribosomal RNA gene, partial sequence; internal transcribed spacer 2, complete sequence;...	102	102	100%	1e-18	100.00%	522	MH547540.1
Ephedra sp. KSR-2018 isolate KSR-0024 5.8S ribosomal RNA gene and internal transcribed spacer 2, partial sequence	102	102	100%	1e-18	100.00%	365	MH732928.1
Ephedra sinica isolate KPS0082A03 internal transcribed spacer 2, partial sequence	102	102	100%	1e-18	100.00%	251	MF096260.1
Ephedra sinica isolate KPS0082A02 internal transcribed spacer 2, partial sequence	102	102	100%	1e-18	100.00%	251	MF096259.1
Ephedra sinica isolate KPS0082A01 internal transcribed spacer 2, partial sequence	102	102	100%	1e-18	100.00%	251	MF096258.1
Ephedra sinica isolate KMS0082A03 internal transcribed spacer 2, partial sequence	102	102	100%	1e-18	100.00%	251	MF096257.1
Ephedra somalensis voucher Thulin 10925A (UPS) internal transcribed spacer 1, partial sequence; 5.8S ribosomal RNA gene and i...	97.1	97.1	100%	6e-17	98.18%	1586	FJ958004.1
Ephedra distachya internal transcribed spacer 2, partial sequence	97.1	97.1	100%	6e-17	98.18%	246	GU186924.1
Ephedra pachyclada voucher (S) C-7844 18S ribosomal RNA gene, partial sequence; internal transcribed spacer 1, 5.8S ribosomal...	97.1	97.1	100%	6e-17	98.18%	1658	AY755779.1
Ephedra distachya voucher (S) 03-684 18S ribosomal RNA gene, partial sequence; internal transcribed spacer 1, 5.8S ribosomal R...	97.1	97.1	100%	6e-17	98.18%	1675	AY755769.1
Ephedra distachya voucher (S) 04-481 18S ribosomal RNA gene, partial sequence; internal transcribed spacer 1, 5.8S ribosomal R...	97.1	97.1	100%	6e-17	98.18%	1675	AY755761.1
Ephedra equisetina voucher (S) 03-928 18S ribosomal RNA gene, partial sequence; internal transcribed spacer 1, 5.8S ribosomal ...	97.1	97.1	100%	6e-17	98.18%	1659	AY755740.1
Ephedra trifurca voucher Reina-G. & Van Devender 96-208 (MEXU) 18S ribosomal RNA gene, partial sequence; internal transcribe...	95.3	95.3	98%	2e-16	98.15%	1657	KP788841.1
Ephedra californica voucher Little & Ochoterena 793 (MEXU) 18S ribosomal RNA gene, partial sequence; internal transcribed spac...	95.3	95.3	98%	2e-16	98.15%	1658	KP788816.1
Ephedra foeminea internal transcribed spacer 1, partial sequence; 5.8S ribosomal RNA gene and internal transcribed spacer 2, co...	95.3	95.3	100%	2e-16	98.18%	1563	KP788808.1
Ephedra trifurca voucher (Mo) 04630447 18S ribosomal RNA gene, partial sequence; internal transcribed spacer 1, 5.8S ribosomal ...	95.3	95.3	98%	2e-16	98.15%	1658	AY755762.1
Ephedra gerardiana voucher (E) E00130259 18S ribosomal RNA gene, partial sequence; internal transcribed spacer 1, 5.8S riboso...	95.3	95.3	100%	2e-16	96.36%	1658	AY755753.1
Ephedra californica voucher (O) 68-154 18S ribosomal RNA gene, partial sequence; internal transcribed spacer 1, 5.8S ribosomal ...	95.3	95.3	98%	2e-16	98.15%	1659	AY755750.1

Figure S2

	Description	Max Score	Total Score	Query Cover	E value	Per. Ident	Acc. Len	Accession
☑	Ephedra likiangensis voucher LJ01 internal transcribed spacer 2, partial sequence	84.2	84.2	100%	3e-13	100.00%	246	MF972970.1
☑	Ephedra sinica voucher CM04 internal transcribed spacer 2, partial sequence	84.2	84.2	100%	3e-13	100.00%	261	MF972969.1
☑	Ephedra sinica voucher CM03 internal transcribed spacer 2, partial sequence	84.2	84.2	100%	3e-13	100.00%	261	MF972968.1
☑	Ephedra sinica voucher CM01 internal transcribed spacer 2, partial sequence	84.2	84.2	100%	3e-13	100.00%	261	MF972967.1
☑	Ephedra sinica voucher CM05 internal transcribed spacer 2, partial sequence	84.2	84.2	100%	3e-13	100.00%	261	MF972966.1
☑	Ephedra sinica voucher CM02 internal transcribed spacer 2, partial sequence	84.2	84.2	100%	3e-13	100.00%	261	MF972965.1
☑	Ephedra major subsp. procera voucher 823-12-2014 5.8S ribosomal RNA gene, partial sequence; internal transcribed spacer 2, co...	84.2	84.2	100%	3e-13	100.00%	331	MG256238.1
☑	Ephedra aphylla voucher SCUI-E13 5.8S ribosomal RNA gene, partial sequence; internal transcribed spacer 2, complete sequence...	84.2	84.2	100%	3e-13	100.00%	356	MG569946.1
☑	Ephedra intermedia isolate XZZMH4 internal transcribed spacer 2, complete sequence	84.2	84.2	100%	3e-13	100.00%	251	MH258183.1
☑	Ephedra intermedia isolate XZZMH3 internal transcribed spacer 2, complete sequence	84.2	84.2	100%	3e-13	100.00%	251	MH258182.1
☑	Ephedra intermedia isolate XZZMH2 internal transcribed spacer 2, complete sequence	84.2	84.2	100%	3e-13	100.00%	251	MH258181.1
☑	Ephedra intermedia isolate XZZMH1 internal transcribed spacer 2, complete sequence	84.2	84.2	100%	3e-13	100.00%	251	MH258180.1
☑	Ephedra intermedia isolate XZZMH internal transcribed spacer 2, complete sequence	84.2	84.2	100%	3e-13	100.00%	251	MH258138.1
☑	Ephedra alata isolate UQU ALP192 5.8S ribosomal RNA gene, partial sequence; internal transcribed spacer 2, complete sequence;...	84.2	84.2	100%	3e-13	100.00%	522	MH547540.1
☑	Ephedra sp. KSR-2018 isolate KSR-0024 5.8S ribosomal RNA gene and internal transcribed spacer 2, partial sequence	84.2	84.2	100%	3e-13	100.00%	365	MH732928.1
☑	Ephedra sinica isolate KPS0082A03 internal transcribed spacer 2, partial sequence	84.2	84.2	100%	3e-13	100.00%	251	MF096260.1
☑	Ephedra sinica isolate KPS0082A02 internal transcribed spacer 2, partial sequence	84.2	84.2	100%	3e-13	100.00%	251	MF096259.1
☑	Ephedra sinica isolate KPS0082A01 internal transcribed spacer 2, partial sequence	84.2	84.2	100%	3e-13	100.00%	251	MF096258.1
☑	Ephedra sinica isolate KMS0082A03 internal transcribed spacer 2, partial sequence	84.2	84.2	100%	3e-13	100.00%	251	MF096257.1
☑	Ephedra distachya isolate 227 internal transcribed spacer 1, partial sequence; 5.8S ribosomal RNA gene and internal transcribed s...	78.7	78.7	100%	1e-11	97.78%	1161	GU968568.1
☑	Ephedra milleri voucher Miller 7667 (E) internal transcribed spacer 1, partial sequence; 5.8S ribosomal RNA gene and internal trans...	78.7	78.7	100%	1e-11	97.78%	1563	FJ958024.1
☑	Ephedra transitoria voucher Collenette 9095B (E) internal transcribed spacer 1, partial sequence; 5.8S ribosomal RNA gene and int...	78.7	78.7	100%	1e-11	97.78%	855	FJ958021.1
☑	Ephedra strobilacea voucher Aellen et Estafandri 2703 (S) internal transcribed spacer 1, partial sequence; 5.8S ribosomal RNA gen...	78.7	78.7	100%	1e-11	97.78%	1599	FJ958018.1
☑	Ephedra sarcocarpa voucher Aellen et Estafandri 2786 (S) internal transcribed spacer 1, partial sequence; 5.8S ribosomal RNA gen...	78.7	78.7	100%	1e-11	97.78%	1601	FJ958017.1
☑	Ephedra distachya voucher Pidopliczka s.n. (UPS) internal transcribed spacer 1, partial sequence; 5.8S ribosomal RNA gene and i...	78.7	78.7	100%	1e-11	97.78%	1603	FJ958013.1
☑	Ephedra somalensis voucher Thulin 10925A (UPS) internal transcribed spacer 1, partial sequence; 5.8S ribosomal RNA gene and i...	78.7	78.7	93%	1e-11	100.00%	1586	FJ958004.1
☑	Ephedra distachya internal transcribed spacer 2, partial sequence	78.7	78.7	100%	1e-11	97.78%	246	GU186924.1
☑	Ephedra pachyclada voucher (S) C-7844 18S ribosomal RNA gene, partial sequence; internal transcribed spacer 1, 5.8S ribosomal...	78.7	78.7	93%	1e-11	100.00%	1658	AY755779.1
☑	Ephedra distachya voucher (S) 03-684 18S ribosomal RNA gene, partial sequence; internal transcribed spacer 1, 5.8S ribosomal R...	78.7	78.7	100%	1e-11	97.78%	1675	AY755769.1
☑	Ephedra trifurca voucher (Mo) 04630447 18S ribosomal RNA gene, partial sequence; internal transcribed spacer 1, 5.8S ribosomal ...	78.7	78.7	100%	1e-11	97.83%	1658	AY755762.1
☑	Ephedra distachya voucher (S) 04-481 18S ribosomal RNA gene, partial sequence; internal transcribed spacer 1, 5.8S ribosomal R...	78.7	78.7	100%	1e-11	97.78%	1675	AY755761.1
☑	Ephedra californica voucher (O) 68-154 18S ribosomal RNA gene, partial sequence; internal transcribed spacer 1, 5.8S ribosomal ...	78.7	78.7	100%	1e-11	97.83%	1659	AY755750.1
☑	Ephedra foeminea internal transcribed spacer 1, partial sequence; 5.8S ribosomal RNA gene and internal transcribed spacer 2, co...	76.8	76.8	100%	5e-11	97.78%	1563	KP788808.1
☑	Ephedra gerardiana voucher (E) E00130259 18S ribosomal RNA gene, partial sequence; internal transcribed spacer 1, 5.8S riboso...	76.8	76.8	100%	5e-11	95.56%	1658	AY755753.1

Figure S3

Description	Max Score	Total Score	Query Cover	E value	Per. Ident	Acc. Len	Accession
Ephedra likiangensis voucher LJ01 internal transcribed spacer 2, partial sequence	65.8	65.8	100%	4e-08	100.00%	246	MF972970.1
Ephedra sinica voucher CM04 internal transcribed spacer 2, partial sequence	65.8	65.8	100%	4e-08	100.00%	261	MF972969.1
Ephedra sinica voucher CM03 internal transcribed spacer 2, partial sequence	65.8	65.8	100%	4e-08	100.00%	261	MF972968.1
Ephedra sinica voucher CM01 internal transcribed spacer 2, partial sequence	65.8	65.8	100%	4e-08	100.00%	261	MF972967.1
Ephedra sinica voucher CM05 internal transcribed spacer 2, partial sequence	65.8	65.8	100%	4e-08	100.00%	261	MF972966.1
Ephedra sinica voucher CM02 internal transcribed spacer 2, partial sequence	65.8	65.8	100%	4e-08	100.00%	261	MF972965.1
Ephedra major subsp. procera voucher 823-12-2014 5.8S ribosomal RNA gene, partial sequence; internal transcribed spacer 2, co...	65.8	65.8	100%	4e-08	100.00%	331	MG256238.1
Ephedra aphylla voucher SCUI-E13 5.8S ribosomal RNA gene, partial sequence; internal transcribed spacer 2, complete sequence...	65.8	65.8	100%	4e-08	100.00%	356	MG569946.1
Ephedra intermedia isolate XZZMH4 internal transcribed spacer 2, complete sequence	65.8	65.8	100%	4e-08	100.00%	251	MH258183.1
Ephedra intermedia isolate XZZMH3 internal transcribed spacer 2, complete sequence	65.8	65.8	100%	4e-08	100.00%	251	MH258182.1
Ephedra intermedia isolate XZZMH2 internal transcribed spacer 2, complete sequence	65.8	65.8	100%	4e-08	100.00%	251	MH258181.1
Ephedra intermedia isolate XZZMH1 internal transcribed spacer 2, complete sequence	65.8	65.8	100%	4e-08	100.00%	251	MH258180.1
Ephedra intermedia isolate XZZMH internal transcribed spacer 2, complete sequence	65.8	65.8	100%	4e-08	100.00%	251	MH258138.1
Ephedra alata isolate UQU ALP192 5.8S ribosomal RNA gene, partial sequence; internal transcribed spacer 2, complete sequence;...	65.8	65.8	100%	4e-08	100.00%	522	MH547540.1
Ephedra sp. KSR-2018 isolate KSR-0024 5.8S ribosomal RNA gene and internal transcribed spacer 2, partial sequence	65.8	65.8	100%	4e-08	100.00%	365	MH732928.1
Ephedra sinica isolate KPS0082A03 internal transcribed spacer 2, partial sequence	65.8	65.8	100%	4e-08	100.00%	251	MF096260.1
Ephedra sinica isolate KPS0082A02 internal transcribed spacer 2, partial sequence	65.8	65.8	100%	4e-08	100.00%	251	MF096259.1
Ephedra sinica isolate KPS0082A01 internal transcribed spacer 2, partial sequence	65.8	65.8	100%	4e-08	100.00%	251	MF096258.1
Ephedra sinica isolate KMS0082A03 internal transcribed spacer 2, partial sequence	65.8	65.8	100%	4e-08	100.00%	251	MF096257.1
Ephedra sinica isolate KMS0082A02 internal transcribed spacer 2, partial sequence	65.8	65.8	100%	4e-08	100.00%	251	MF096256.1
Ephedra gerardiana voucher (K) 10141 18S ribosomal RNA gene, partial sequence; internal transcribed spacer 1, 5.8S ribosomal R...	65.8	65.8	100%	4e-08	100.00%	1658	AY755745.1
Ephedra frustillata voucher (S) 04-482 18S ribosomal RNA gene, partial sequence; internal transcribed spacer 1, 5.8S ribosomal R...	65.8	65.8	100%	4e-08	100.00%	1659	AY755743.1
Ephedra intermedia voucher (S) 03-925 18S ribosomal RNA gene, partial sequence; internal transcribed spacer 1, 5.8S ribosomal ...	65.8	65.8	100%	4e-08	100.00%	1677	AY755741.1
Ephedra equisetina voucher (S) 03-928 18S ribosomal RNA gene, partial sequence; internal transcribed spacer 1, 5.8S ribosomal ...	65.8	65.8	100%	4e-08	100.00%	1659	AY755740.1
Ephedra likiangensis voucher (S) 03-926 18S ribosomal RNA gene, partial sequence; internal transcribed spacer 1, 5.8S ribosomal ...	65.8	65.8	100%	4e-08	100.00%	1662	AY755739.1
Ephedra rhytidosperma 18S ribosomal RNA gene, partial sequence; internal transcribed spacer 1, 5.8S ribosomal RNA gene, and i...	65.8	65.8	100%	4e-08	100.00%	1590	DQ028782.2
Ephedra przewalskii voucher Kamp03071110 internal transcribed spacer 2, partial sequence	65.8	65.8	100%	4e-08	100.00%	246	AY730605.1
Ephedra pachyclada voucher SCUI-E11 5.8S ribosomal RNA gene, partial sequence; internal transcribed spacer 2, complete seque...	63.9	63.9	97%	1e-07	100.00%	357	MG569945.1
Ephedra somalensis voucher Thulin 10925A (UPS) internal transcribed spacer 1, partial sequence; 5.8S ribosomal RNA gene and i...	63.9	63.9	97%	1e-07	100.00%	1586	FJ958004.1
Ephedra pachyclada voucher (S) C-7844 18S ribosomal RNA gene, partial sequence; internal transcribed spacer 1, 5.8S ribosomal...	63.9	63.9	97%	1e-07	100.00%	1658	AY755779.1
Ephedra minuta voucher (S) 03-930 18S ribosomal RNA gene, partial sequence; internal transcribed spacer 1, 5.8S ribosomal RNA...	62.1	62.1	94%	5e-07	100.00%	1662	AY755742.1
Ephedra gerardiana voucher (E) E00130259 18S ribosomal RNA gene, partial sequence; internal transcribed spacer 1, 5.8S riboso...	60.2	60.2	91%	2e-06	100.00%	1658	AY755753.1

Figure S4

Description	Max Score	Total Score	Query Cover	E value	Per. Ident	Acc. Len	Accession
Ephedra likiangensis voucher LJ01 internal transcribed spacer 2, partial sequence	60.0	60.0	100%	3e-06	100.00%	246	MF972970.1
Ephedra sinica voucher CM04 internal transcribed spacer 2, partial sequence	60.0	60.0	100%	3e-06	100.00%	261	MF972969.1
Ephedra sinica voucher CM03 internal transcribed spacer 2, partial sequence	60.0	60.0	100%	3e-06	100.00%	261	MF972968.1
Ephedra sinica voucher CM01 internal transcribed spacer 2, partial sequence	60.0	60.0	100%	3e-06	100.00%	261	MF972967.1
Ephedra sinica voucher CM05 internal transcribed spacer 2, partial sequence	60.0	60.0	100%	3e-06	100.00%	261	MF972966.1
Ephedra sinica voucher CM02 internal transcribed spacer 2, partial sequence	60.0	60.0	100%	3e-06	100.00%	261	MF972965.1
Ephedra major subsp. procera voucher 823-12-2014 5.8S ribosomal RNA gene, partial sequence; internal transcribed sp...	60.0	60.0	100%	3e-06	100.00%	331	MG256238.1
Ephedra aphylla voucher SCUI-E13 5.8S ribosomal RNA gene, partial sequence; internal transcribed spacer 2, complete ...	60.0	60.0	100%	3e-06	100.00%	356	MG569946.1
Ephedra intermedia isolate XZZMH4 internal transcribed spacer 2, complete sequence	60.0	60.0	100%	3e-06	100.00%	251	MH258183.1
Ephedra intermedia isolate XZZMH3 internal transcribed spacer 2, complete sequence	60.0	60.0	100%	3e-06	100.00%	251	MH258182.1
Ephedra intermedia isolate XZZMH2 internal transcribed spacer 2, complete sequence	60.0	60.0	100%	3e-06	100.00%	251	MH258181.1
Ephedra intermedia isolate XZZMH1 internal transcribed spacer 2, complete sequence	60.0	60.0	100%	3e-06	100.00%	251	MH258180.1
Ephedra intermedia isolate XZZMH internal transcribed spacer 2, complete sequence	60.0	60.0	100%	3e-06	100.00%	251	MH258138.1
Ephedra alata isolate UQU ALP192 5.8S ribosomal RNA gene, partial sequence; internal transcribed spacer 2, complete ...	60.0	60.0	100%	3e-06	100.00%	522	MH547540.1
Ephedra sp. KSR-2018 isolate KSR-0024 5.8S ribosomal RNA gene and internal transcribed spacer 2, partial sequence	60.0	60.0	100%	3e-06	100.00%	365	MH732928.1
Ephedra sinica isolate KPS0082A03 internal transcribed spacer 2, partial sequence	60.0	60.0	100%	3e-06	100.00%	251	MF096260.1
Ephedra sinica isolate KPS0082A02 internal transcribed spacer 2, partial sequence	60.0	60.0	100%	3e-06	100.00%	251	MF096259.1
Ephedra sinica isolate KPS0082A01 internal transcribed spacer 2, partial sequence	60.0	60.0	100%	3e-06	100.00%	251	MF096258.1
Ephedra sinica isolate KMS0082A03 internal transcribed spacer 2, partial sequence	60.0	60.0	100%	3e-06	100.00%	251	MF096257.1
Ephedra przewalskii voucher Kamp03071110 internal transcribed spacer 2, partial sequence	60.0	60.0	100%	3e-06	100.00%	246	AY730605.1
Ephedra pachyclada voucher SCUI-E11 5.8S ribosomal RNA gene, partial sequence; internal transcribed spacer 2, compl...	58.0	58.0	96%	1e-05	100.00%	357	MG569945.1
Ephedra somalensis voucher Thulin 10925A (UPS) internal transcribed spacer 1, partial sequence; 5.8S ribosomal RNA g...	58.0	58.0	96%	1e-05	100.00%	1586	FJ958004.1
Ephedra pachyclada voucher (S) C-7844 18S ribosomal RNA gene, partial sequence; internal transcribed spacer 1, 5.8S ...	58.0	58.0	96%	1e-05	100.00%	1658	AY755779.1
Ephedra triandra voucher Ickert-Bond 1227 (ASU) 18S ribosomal RNA gene, partial sequence; internal transcribed space...	54.0	54.0	90%	2e-04	100.00%	1649	KP788840.1
Ephedra foeminea internal transcribed spacer 1, partial sequence; 5.8S ribosomal RNA gene and internal transcribed spa...	54.0	54.0	90%	2e-04	100.00%	1563	KP788808.1
Ephedra foeminea voucher Osvald 26068 (UPS) internal transcribed spacer 1, partial sequence; 5.8S ribosomal RNA gen...	54.0	54.0	100%	2e-04	96.67%	1564	FJ958007.1
Ephedra andina voucher (K) 10140 18S ribosomal RNA gene, partial sequence; internal transcribed spacer 1, 5.8S riboso...	54.0	54.0	100%	2e-04	96.67%	1659	AY755744.1
Ephedra major subsp. procera voucher H. Freitag 26.636 internal transcribed spacer 2, complete sequence	52.0	52.0	86%	9e-04	100.00%	246	JQ726590.1
Ephedra distachya isolate 227 internal transcribed spacer 1, partial sequence; 5.8S ribosomal RNA gene and internal tran...	52.0	52.0	86%	9e-04	100.00%	1161	GU968568.1
Ephedra milleri voucher Miller 7667 (E) internal transcribed spacer 1, partial sequence; 5.8S ribosomal RNA gene and int...	52.0	52.0	100%	9e-04	96.67%	1563	FJ958024.1
Ephedra transitoria voucher Collenette 9095B (E) internal transcribed spacer 1, partial sequence; 5.8S ribosomal RNA ge...	52.0	52.0	86%	9e-04	100.00%	855	FJ958021.1
Ephedra strobilacea voucher Aellen et Estafandri 2703 (S) internal transcribed spacer 1, partial sequence; 5.8S ribosomal...	52.0	52.0	86%	9e-04	100.00%	1599	FJ958018.1
Ephedra sarcocarpa voucher Aellen et Estafandri 2786 (S) internal transcribed spacer 1, partial sequence; 5.8S ribosoma...	52.0	52.0	86%	9e-04	100.00%	1601	FJ958017.1
Ephedra distachya voucher Pidopliczka s.n. (UPS) internal transcribed spacer 1, partial sequence; 5.8S ribosomal RNA g...	52.0	52.0	86%	9e-04	100.00%	1603	FJ958013.1
Ephedra distachya internal transcribed spacer 2, partial sequence	52.0	52.0	86%	9e-04	100.00%	246	GU186924.1
Ephedra distachya voucher (S) 03-684 18S ribosomal RNA gene, partial sequence; internal transcribed spacer 1, 5.8S rib...	52.0	52.0	86%	9e-04	100.00%	1675	AY755769.1

Figure S5

Description	Max Score	Total Score	Query Cover	E value	Per. Ident	Acc. Len	Accession
Ephedra likiangensis voucher LJ01 internal transcribed spacer 2, partial sequence	54.0	54.0	100%	1e-04	100.00%	246	MF972970.1
Ephedra sinica voucher CM04 internal transcribed spacer 2, partial sequence	54.0	54.0	100%	1e-04	100.00%	261	MF972969.1
Ephedra sinica voucher CM03 internal transcribed spacer 2, partial sequence	54.0	54.0	100%	1e-04	100.00%	261	MF972968.1
Ephedra sinica voucher CM01 internal transcribed spacer 2, partial sequence	54.0	54.0	100%	1e-04	100.00%	261	MF972967.1
Ephedra sinica voucher CM05 internal transcribed spacer 2, partial sequence	54.0	54.0	100%	1e-04	100.00%	261	MF972966.1
Ephedra sinica voucher CM02 internal transcribed spacer 2, partial sequence	54.0	54.0	100%	1e-04	100.00%	261	MF972965.1
Ephedra major subsp. procera voucher 823-12-2014 5.8S ribosomal RNA gene, partial sequence; internal transcribed sp...	54.0	54.0	100%	1e-04	100.00%	331	MG256238.1
Ephedra aphylla voucher SCUI-E13 5.8S ribosomal RNA gene, partial sequence; internal transcribed spacer 2, complete ...	54.0	54.0	100%	1e-04	100.00%	356	MG569946.1
Ephedra pachyclada voucher SCUI-E11 5.8S ribosomal RNA gene, partial sequence; internal transcribed spacer 2, compl...	54.0	54.0	100%	1e-04	100.00%	357	MG569945.1
Ephedra intermedia isolate XZZMH4 internal transcribed spacer 2, complete sequence	54.0	54.0	100%	1e-04	100.00%	251	MH258183.1
Ephedra intermedia isolate XZZMH3 internal transcribed spacer 2, complete sequence	54.0	54.0	100%	1e-04	100.00%	251	MH258182.1
Ephedra intermedia isolate XZZMH2 internal transcribed spacer 2, complete sequence	54.0	54.0	100%	1e-04	100.00%	251	MH258181.1
Ephedra intermedia isolate XZZMH1 internal transcribed spacer 2, complete sequence	54.0	54.0	100%	1e-04	100.00%	251	MH258180.1
Ephedra intermedia isolate XZZMH internal transcribed spacer 2, complete sequence	54.0	54.0	100%	1e-04	100.00%	251	MH258138.1
Ephedra alata isolate UQU ALP192 5.8S ribosomal RNA gene, partial sequence; internal transcribed spacer 2, complete ...	54.0	54.0	100%	1e-04	100.00%	522	MH547540.1
Ephedra sp. KSR-2018 isolate KSR-0024 5.8S ribosomal RNA gene and internal transcribed spacer 2, partial sequence	54.0	54.0	100%	1e-04	100.00%	365	MH732928.1
Ephedra sinica isolate KPS0082A03 internal transcribed spacer 2, partial sequence	54.0	54.0	100%	1e-04	100.00%	251	MF096260.1
Ephedra sinica isolate KPS0082A02 internal transcribed spacer 2, partial sequence	54.0	54.0	100%	1e-04	100.00%	251	MF096259.1
Ephedra sinica isolate KPS0082A01 internal transcribed spacer 2, partial sequence	54.0	54.0	100%	1e-04	100.00%	251	MF096258.1
Ephedra sinica isolate KMS0082A03 internal transcribed spacer 2, partial sequence	54.0	54.0	100%	1e-04	100.00%	251	MF096257.1
Ephedra frustillata voucher (S) 04-482 18S ribosomal RNA gene, partial sequence; internal transcribed spacer 1, 5.8S rib...	54.0	54.0	100%	1e-04	100.00%	1659	AY755743.1
Ephedra minuta voucher (S) 03-930 18S ribosomal RNA gene, partial sequence; internal transcribed spacer 1, 5.8S ribos...	54.0	54.0	100%	1e-04	100.00%	1662	AY755742.1
Ephedra intermedia voucher (S) 03-925 18S ribosomal RNA gene, partial sequence; internal transcribed spacer 1, 5.8S ri...	54.0	54.0	100%	1e-04	100.00%	1677	AY755741.1
Ephedra equisetina voucher (S) 03-928 18S ribosomal RNA gene, partial sequence; internal transcribed spacer 1, 5.8S ri...	54.0	54.0	100%	1e-04	100.00%	1659	AY755740.1
Ephedra likiangensis voucher (S) 03-926 18S ribosomal RNA gene, partial sequence; internal transcribed spacer 1, 5.8S ...	54.0	54.0	100%	1e-04	100.00%	1662	AY755739.1
Ephedra rhytidosperma 18S ribosomal RNA gene, partial sequence; internal transcribed spacer 1, 5.8S ribosomal RNA g...	54.0	54.0	100%	1e-04	100.00%	1590	DQ028782.2
Ephedra przewalskii voucher Kamp03071110 internal transcribed spacer 2, partial sequence	54.0	54.0	100%	1e-04	100.00%	246	AY730605.1
Ephedra triandra voucher Ickert-Bond 1227 (ASU) 18S ribosomal RNA gene, partial sequence; internal transcribed space...	52.0	52.0	96%	6e-04	100.00%	1649	KP788840.1
Ephedra foeminea internal transcribed spacer 1, partial sequence; 5.8S ribosomal RNA gene and internal transcribed spa...	52.0	52.0	96%	6e-04	100.00%	1563	KP788808.1
Ephedra major subsp. procera voucher H. Freitag 26.636 internal transcribed spacer 2, complete sequence	50.1	50.1	92%	0.002	100.00%	246	JQ726590.1
Ephedra distachya isolate 227 internal transcribed spacer 1, partial sequence; 5.8S ribosomal RNA gene and internal tran...	50.1	50.1	92%	0.002	100.00%	1161	GU968568.1
Ephedra transitoria voucher Collenette 9095B (E) internal transcribed spacer 1, partial sequence; 5.8S ribosomal RNA ge...	50.1	50.1	92%	0.002	100.00%	855	FJ958021.1
Ephedra strobilacea voucher Aellen et Estafandri 2703 (S) internal transcribed spacer 1, partial sequence; 5.8S ribosomal...	50.1	50.1	92%	0.002	100.00%	1599	FJ958018.1
Ephedra sarcocarpa voucher Aellen et Estafandri 2786 (S) internal transcribed spacer 1, partial sequence; 5.8S ribosoma...	50.1	50.1	92%	0.002	100.00%	1601	FJ958017.1
Ephedra distachya voucher Pidopliczka s.n. (UPS) internal transcribed spacer 1, partial sequence; 5.8S ribosomal RNA g...	50.1	50.1	92%	0.002	100.00%	1603	FJ958013.1
Ephedra foeminea voucher Osvald 26068 (UPS) internal transcribed spacer 1, partial sequence; 5.8S ribosomal RNA gen...	50.1	50.1	92%	0.002	100.00%	1564	FJ958007.1
Ephedra distachya internal transcribed spacer 2, partial sequence	50.1	50.1	92%	0.002	100.00%	246	GU186924.1
Ephedra distachya voucher (S) 03-684 18S ribosomal RNA gene, partial sequence; internal transcribed spacer 1, 5.8S rib...	50.1	50.1	92%	0.002	100.00%	1675	AY755769.1
Ephedra distachya voucher (S) 04-481 18S ribosomal RNA gene, partial sequence; internal transcribed spacer 1, 5.8S rib...	50.1	50.1	92%	0.002	100.00%	1675	AY755761.1
Ephedra andina voucher (K) 10140 18S ribosomal RNA gene, partial sequence; internal transcribed spacer 1, 5.8S riboso...	48.1	48.1	100%	0.009	96.30%	1659	AY755744.1
Ephedra milleri voucher Miller 7667 (E) internal transcribed spacer 1, partial sequence; 5.8S ribosomal RNA gene and int...	46.1	46.1	100%	0.035	96.30%	1563	FJ958024.1
Streptomyces sp. TLI_053 genome assembly, chromosome: I	44.1	374	85%	0.14	100.00%	9900053	LT629775.1
Colchicum chlorobasis voucher C4225 internal transcribed spacer 1 and 5.8S ribosomal RNA gene, partial sequence	44.1	44.1	81%	0.14	100.00%	202	MF596365.1

Figure S6

	Description	Max Score	Total Score	Query Cover	E value	Per. Ident	Acc. Len	Accession
☑	Nocardioides cynanchi strain SB3-45 chromosome, complete genome	40.1	163	100%	0.72	100.00%	3998545	CP044344.1
☑	Pseudomonas thivervalensis strain PLM3 genome	40.1	40.1	100%	0.72	100.00%	6591188	CP022202.1
☑	Pseudomonas thivervalensis strain SC5 genome	40.1	40.1	100%	0.72	100.00%	6592350	CP022201.1
☑	Streptomyces sp. TLI_053 genome assembly, chromosome: I	40.1	636	100%	0.72	100.00%	9900053	LT629775.1
☑	Pseudomonas thivervalensis strain BS3779 genome assembly, chromosome: I	40.1	40.1	100%	0.72	100.00%	6604078	LT629691.1
☑	Micromonospora coxensis strain DSM 45161 genome assembly, chromosome: I	40.1	686	100%	0.72	100.00%	6769793	LT607753.1
☑	Halopenitus persicus DNA, complete genome, strain: CBA1233	40.1	197	100%	0.72	100.00%	2967917	AP017558.1
☑	Mycobacterium riyadhense strain NTM chromosome	40.1	102	100%	0.72	100.00%	6772223	CP045092.1
☑	Curtobacterium sp. YC1 chromosome, complete genome	40.1	130	100%	0.72	100.00%	3301309	CP066341.1
☑	Salinispora tropica CNB-440, complete genome	40.1	191	100%	0.72	100.00%	5183331	CP000667.1
☑	XXX	40.1	40.1	100%	0.72	100.00%	46394	LR589107.1
☑	XXX	40.1	40.1	100%	0.72	100.00%	46394	LR589013.1
☑	Colchicum chlorobasis voucher C4225 internal transcribed spacer 1 and 5.8S ribosomal RNA gene, partial sequence	40.1	40.1	100%	0.72	100.00%	202	MF596365.1
☑	Ephedra likiangensis voucher LJ01 internal transcribed spacer 2, partial sequence	40.1	40.1	100%	0.72	100.00%	246	MF972970.1
☑	Ephedra sinica voucher CM04 internal transcribed spacer 2, partial sequence	40.1	40.1	100%	0.72	100.00%	261	MF972969.1
☑	Ephedra sinica voucher CM03 internal transcribed spacer 2, partial sequence	40.1	40.1	100%	0.72	100.00%	261	MF972968.1
☑	Ephedra sinica voucher CM01 internal transcribed spacer 2, partial sequence	40.1	40.1	100%	0.72	100.00%	261	MF972967.1
☑	Ephedra sinica voucher CM05 internal transcribed spacer 2, partial sequence	40.1	40.1	100%	0.72	100.00%	261	MF972966.1
☑	Ephedra sinica voucher CM02 internal transcribed spacer 2, partial sequence	40.1	40.1	100%	0.72	100.00%	261	MF972965.1
☑	Uncultured organism clone KBTEX_76 genomic sequence	40.1	40.1	100%	0.72	100.00%	14869	MN079151.1
☑	Ephedra major subsp. procera voucher 823-12-2014 5.8S ribosomal RNA gene, partial sequence; internal transcribed sp...	40.1	40.1	100%	0.72	100.00%	331	MG256238.1
☑	Ephedra aphylla voucher SCUI-E13 5.8S ribosomal RNA gene, partial sequence; internal transcribed spacer 2, complete ...	40.1	40.1	100%	0.72	100.00%	356	MG569946.1
☑	Ephedra pachyclada voucher SCUI-E11 5.8S ribosomal RNA gene, partial sequence; internal transcribed spacer 2, compl...	40.1	40.1	100%	0.72	100.00%	357	MG569945.1
☑	Ephedra intermedia isolate XZZMH4 internal transcribed spacer 2, complete sequence	40.1	40.1	100%	0.72	100.00%	251	MH258183.1
☑	Ephedra intermedia isolate XZZMH3 internal transcribed spacer 2, complete sequence	40.1	40.1	100%	0.72	100.00%	251	MH258182.1
☑	Ephedra intermedia isolate XZZMH2 internal transcribed spacer 2, complete sequence	40.1	40.1	100%	0.72	100.00%	251	MH258181.1
☑	Ephedra intermedia isolate XZZMH1 internal transcribed spacer 2, complete sequence	40.1	40.1	100%	0.72	100.00%	251	MH258180.1

Figure S7

Description	Max Score	Total Score	Query Cover	E value	Per. Ident	Acc. Len	Accession
Ephedra likiangensis voucher LJ01 internal transcribed spacer 2, partial sequence	46.1	46.1	100%	0.018	100.00%	246	MF972970.1
Ephedra sinica voucher CM04 internal transcribed spacer 2, partial sequence	46.1	46.1	100%	0.018	100.00%	261	MF972969.1
Ephedra sinica voucher CM03 internal transcribed spacer 2, partial sequence	46.1	46.1	100%	0.018	100.00%	261	MF972968.1
Ephedra sinica voucher CM01 internal transcribed spacer 2, partial sequence	46.1	46.1	100%	0.018	100.00%	261	MF972967.1
Ephedra sinica voucher CM05 internal transcribed spacer 2, partial sequence	46.1	46.1	100%	0.018	100.00%	261	MF972966.1
Ephedra sinica voucher CM02 internal transcribed spacer 2, partial sequence	46.1	46.1	100%	0.018	100.00%	261	MF972965.1
Ephedra major subsp. procera voucher 823-12-2014 5.8S ribosomal RNA gene, partial sequence; internal transcribed sp...	46.1	46.1	100%	0.018	100.00%	331	MG256238.1
Ephedra aphylla voucher SCUI-E13 5.8S ribosomal RNA gene, partial sequence; internal transcribed spacer 2, complete ...	46.1	46.1	100%	0.018	100.00%	356	MG569946.1
Ephedra pachyclada voucher SCUI-E11 5.8S ribosomal RNA gene, partial sequence; internal transcribed spacer 2, compl...	46.1	46.1	100%	0.018	100.00%	357	MG569945.1
Ephedra intermedia isolate XZZMH4 internal transcribed spacer 2, complete sequence	46.1	46.1	100%	0.018	100.00%	251	MH258183.1
Ephedra intermedia isolate XZZMH3 internal transcribed spacer 2, complete sequence	46.1	46.1	100%	0.018	100.00%	251	MH258182.1
Ephedra intermedia isolate XZZMH2 internal transcribed spacer 2, complete sequence	46.1	46.1	100%	0.018	100.00%	251	MH258181.1
Ephedra intermedia isolate XZZMH1 internal transcribed spacer 2, complete sequence	46.1	46.1	100%	0.018	100.00%	251	MH258180.1
Ephedra intermedia isolate XZZMH internal transcribed spacer 2, complete sequence	46.1	46.1	100%	0.018	100.00%	251	MH258138.1
Ephedra alata isolate UQU ALP192 5.8S ribosomal RNA gene, partial sequence; internal transcribed spacer 2, complete ...	46.1	46.1	100%	0.018	100.00%	522	MH547540.1
Ephedra sp. KSR-2018 isolate KSR-0024 5.8S ribosomal RNA gene and internal transcribed spacer 2, partial sequence	46.1	46.1	100%	0.018	100.00%	365	MH732928.1
Ephedra sinica isolate KPS0082A03 internal transcribed spacer 2, partial sequence	46.1	46.1	100%	0.018	100.00%	251	MF096260.1
Ephedra sinica isolate KPS0082A02 internal transcribed spacer 2, partial sequence	46.1	46.1	100%	0.018	100.00%	251	MF096259.1
Ephedra sinica isolate KPS0082A01 internal transcribed spacer 2, partial sequence	46.1	46.1	100%	0.018	100.00%	251	MF096258.1
Ephedra sinica isolate KMS0082A03 internal transcribed spacer 2, partial sequence	46.1	46.1	100%	0.018	100.00%	251	MF096257.1
Ephedra sinica isolate KMS0082A02 internal transcribed spacer 2, partial sequence	46.1	46.1	100%	0.018	100.00%	251	MF096256.1
Ephedra intermedia isolate KMS0081A03 internal transcribed spacer 2, partial sequence	46.1	46.1	100%	0.018	100.00%	251	MF096255.1
Ephedra intermedia isolate KMS0081A02 internal transcribed spacer 2, partial sequence	46.1	46.1	100%	0.018	100.00%	251	MF096254.1
Ephedra intermedia isolate KMS0081A01 internal transcribed spacer 2, partial sequence	46.1	46.1	100%	0.018	100.00%	251	MF096253.1
Ephedra sinica isolate KMS0082A01 internal transcribed spacer 2, partial sequence	46.1	46.1	100%	0.018	100.00%	251	MF096252.1
Ephedra intermedia isolate KPS0081A02 internal transcribed spacer 2, partial sequence	46.1	46.1	100%	0.018	100.00%	251	MF096251.1
Ephedra intermedia isolate KPS0081A03 internal transcribed spacer 2, partial sequence	46.1	46.1	100%	0.018	100.00%	251	MF096250.1
Ephedra intermedia isolate KPS0081A01 internal transcribed spacer 2, partial sequence	46.1	46.1	100%	0.018	100.00%	251	MF096249.1
Ephedra equisetina isolate KPS0080A03 internal transcribed spacer 2, partial sequence	46.1	46.1	100%	0.018	100.00%	251	MF096248.1
Ephedra equisetina isolate KPS0080A01 internal transcribed spacer 2, partial sequence	46.1	46.1	100%	0.018	100.00%	251	MF096247.1
Ephedra equisetina isolate KMS0080A02 internal transcribed spacer 2, partial sequence	46.1	46.1	100%	0.018	100.00%	251	MF096246.1
Ephedra equisetina isolate KMS0080A01 internal transcribed spacer 2, partial sequence	46.1	46.1	100%	0.018	100.00%	251	MF096245.1
Ephedra equisetina isolate KPS0080A02 internal transcribed spacer 2, partial sequence	46.1	46.1	100%	0.018	100.00%	251	MF096244.1
Ephedra equisetina isolate KMS0080A03 internal transcribed spacer 2, partial sequence	46.1	46.1	100%	0.018	100.00%	251	MF096243.1
Ephedra saxatilis 5.8S ribosomal RNA gene, partial sequence; internal transcribed spacer 2, complete sequence; and 26...	46.1	46.1	100%	0.018	100.00%	370	MF785606.1
Ephedra intermedia voucher YC0347MT07 internal transcribed spacer 2, partial sequence	46.1	46.1	100%	0.018	100.00%	251	KX779088.1
Ephedra intermedia voucher YC0347MT21 internal transcribed spacer 2, partial sequence	46.1	46.1	100%	0.018	100.00%	251	KX779087.1
Ephedra regeliana voucher YC0715MT03 internal transcribed spacer 2, partial sequence	46.1	46.1	100%	0.018	100.00%	251	KX779086.1
Ephedra intermedia var. tibetica voucher YC0710MT04 internal transcribed spacer 2, partial sequence	46.1	46.1	100%	0.018	100.00%	251	KX779085.1
Ephedra distachya voucher YC0709MT01 internal transcribed spacer 2, partial sequence	46.1	46.1	100%	0.018	100.00%	251	KX779084.1
Ephedra lomatolepis voucher YC0714MT01 internal transcribed spacer 2, partial sequence	46.1	46.1	100%	0.018	100.00%	251	KX779083.1
Ephedra sinica voucher (S) CRetoh 18S ribosomal RNA gene, partial sequence; internal transcribed spacer 1, 5.8S ribos...	46.1	46.1	100%	0.018	100.00%	1676	AY755749.1
Ephedra frustillata voucher (K) 10218 18S ribosomal RNA gene, partial sequence; internal transcribed spacer 1, 5.8S ribo...	46.1	46.1	100%	0.018	100.00%	1659	AY755748.1
Ephedra sinica voucher (K) 10143 18S ribosomal RNA gene, partial sequence; internal transcribed spacer 1, 5.8S riboso...	46.1	46.1	100%	0.018	100.00%	1677	AY755747.1
Ephedra monosperma voucher (K) 10142 18S ribosomal RNA gene, partial sequence; internal transcribed spacer 1, 5.8S ...	46.1	46.1	100%	0.018	100.00%	1677	AY755746.1
Ephedra gerardiana voucher (K) 10141 18S ribosomal RNA gene, partial sequence; internal transcribed spacer 1, 5.8S ri...	46.1	46.1	100%	0.018	100.00%	1658	AY755745.1
Ephedra frustillata voucher (S) 04-482 18S ribosomal RNA gene, partial sequence; internal transcribed spacer 1, 5.8S rib...	46.1	46.1	100%	0.018	100.00%	1659	AY755743.1
Ephedra minuta voucher (S) 03-930 18S ribosomal RNA gene, partial sequence; internal transcribed spacer 1, 5.8S ribos...	46.1	46.1	100%	0.018	100.00%	1662	AY755742.1
Ephedra intermedia voucher (S) 03-925 18S ribosomal RNA gene, partial sequence; internal transcribed spacer 1, 5.8S ri...	46.1	46.1	100%	0.018	100.00%	1677	AY755741.1
Ephedra equisetina voucher (S) 03-928 18S ribosomal RNA gene, partial sequence; internal transcribed spacer 1, 5.8S ri...	46.1	46.1	100%	0.018	100.00%	1659	AY755740.1
Ephedra likiangensis voucher (S) 03-926 18S ribosomal RNA gene, partial sequence; internal transcribed spacer 1, 5.8S ...	46.1	46.1	100%	0.018	100.00%	1662	AY755739.1
Ephedra rhytidosperma 18S ribosomal RNA gene, partial sequence; internal transcribed spacer 1, 5.8S ribosomal RNA g...	46.1	46.1	100%	0.018	100.00%	1590	DQ028782.2
Ephedra przewalskii voucher Kamp03071110 internal transcribed spacer 2, partial sequence	46.1	46.1	100%	0.018	100.00%	246	AY730605.1
Brevibacterium sp. o2 chromosome, complete genome	42.1	140	91%	0.27	100.00%	4253182	CP050154.1
Colchicum chlorobasis voucher C4225 internal transcribed spacer 1 and 5.8S ribosomal RNA gene, partial sequence	42.1	42.1	91%	0.27	100.00%	202	MF596365.1
Brevibacterium aurantiacum strain SMQ-1419 chromosome, complete genome	42.1	42.1	91%	0.27	100.00%	4038634	CP025333.1
Brevibacterium aurantiacum strain SMQ-1421 chromosome, complete genome	42.1	74.3	91%	0.27	100.00%	4085228	CP025332.1
Brevibacterium aurantiacum strain SMQ-1418 chromosome, complete genome	42.1	42.1	91%	0.27	100.00%	4193206	CP025331.1
Brevibacterium aurantiacum strain SMQ-1420 chromosome, complete genome	42.1	74.3	91%	0.27	100.00%	4328723	CP025334.1
Brevibacterium aurantiacum strain SMQ-1417 chromosome, complete genome	42.1	74.3	91%	0.27	100.00%	4424341	CP025330.1
Deinococcus actinosclerus strain SJTR chromosome, complete genome	42.1	42.1	91%	0.27	100.00%	3315586	CP029774.1
Streptomyces sp. TLI_053 genome assembly, chromosome: I	42.1	307	95%	0.27	100.00%	9900053	LT629775.1
Brevibacterium aurantiacum strain SMQ-1335 chromosome, complete genome	42.1	74.3	91%	0.27	100.00%	4209935	CP017150.1
Curtobacterium sp. YC1 chromosome, complete genome	42.1	42.1	91%	0.27	100.00%	3301309	CP066341.1
